# Phenotypic effects from the expression of a deregulated *AtGAD1* transgene and GABA pathway suppression mutants in maize

**DOI:** 10.1371/journal.pone.0259365

**Published:** 2021-12-06

**Authors:** Rajani M. S, Mohamed F. Bedair, Hong Li, Stephen M. G. Duff

**Affiliations:** Bayer US, Chesterfield, MO, United States of America; SativaGen, UNITED STATES

## Abstract

Glutamate decarboxylase (GAD; EC 4.1.1.15) catalyzes the irreversible decarboxylation of glutamate to produce γ-aminobutyric acid (GABA); a ubiquitous non-protein amino acid involved in the regulation of several aspects of plant metabolism and physiology. To study the function of GAD and GABA in maize, we have; 1) introduced native and deregulated forms of *AtGAD1* into maize with the intent of increasing the synthesis of GABA and 2) introduced constructs into maize designed to suppress the activity of several GABA shunt, GABA transport and GABA pathway genes. Maize plants expressing the deregulated AtGAD1 exhibit a severe chlorosis and retarded growth phenotype and have high levels of GABA, and Ca^++^/CaM-independent GAD activity. Plants expressing the suppression constructs for GABA biosynthetic and transport pathway genes had no observable phenotype whereas a knockout of GABA catabolic pathway genes led to growth and developmental defects under standard growth conditions. The implications of this study to our understanding of the action and function of GABA and GAD in crops are discussed.

## Introduction

**Fig 1:**
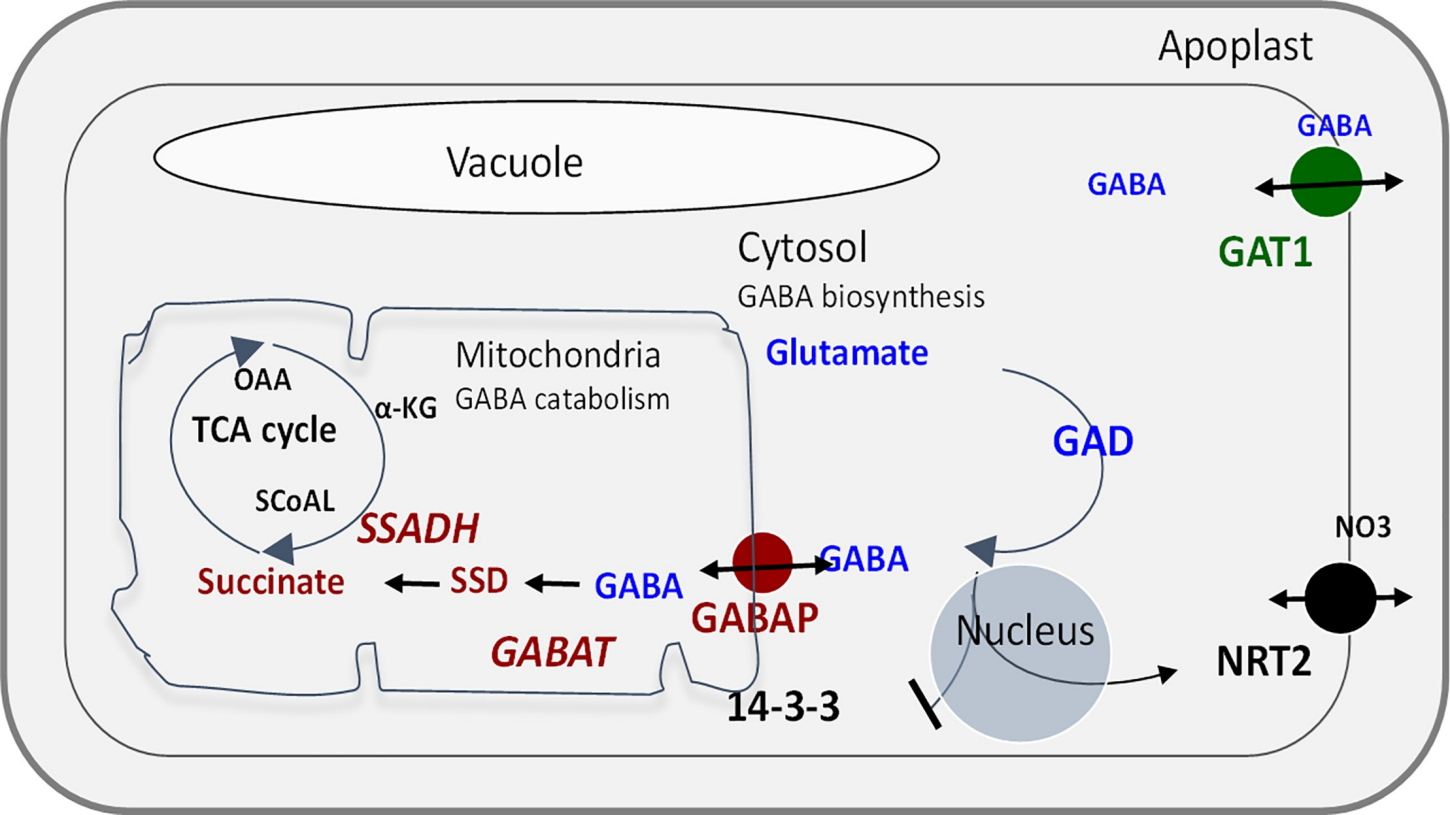
GABA biosynthesis, transport, and metabolism. All abbreviations are defined in Table 1 except, OAA, 2-oxaloacetate; NRT2, nitrate transporter.

Glutamate decarboxylase (GAD; EC 4.1.1.15) catalyzes the irreversible decarboxylation of glutamate to produce GABA (γ-aminobutyric acid). GABA is a ubiquitous non-protein amino acid with a well-known role as an inhibitory neurotransmitter [1]. In plants, GABA is rapidly synthesized in response to biotic and abiotic stresses and catabolized through a short pathway known as the GABA shunt (Fig 1). GABA levels in plants are thus determined by biosynthesis through GAD, catabolism and the GABA shunt, aminotransferases, and several GABA specific transporters on the mitochondrial and plasma membranes [2].

GABA and the GABA shunt are involved in many aspects of plant metabolism and physiology, including the regulation of cytosolic pH, carbon fluxes into the TCA cycle, nitrogen metabolism, oxidative stress tolerance, osmoregulation and signaling [3]. Recently, roles for GAD and GABA in aluminum stress [4], respiration [5] and photosynthesis [6] have been described. There is growing evidence in plants that the GABA shunt plays a major role in primary C/N metabolism [7–10]. A general survey of the literature suggests that the roles of GABA as a signal and metabolite may be coordinated [5]. Towards an integrated theory of GABA in plants, it has been suggested that various roles of GABA in plants might be tightly coupled and functionally linked, its roles as a signal and metabolite facilitating a common purpose [11, 12].

In Arabidopsis, there are five members of the GAD gene family. GAD1, 2 and 4 possess a Ca^++^/CAM binding domain and are probably regulated by Ca^++^/CAM [13] while based on sequence evidence GAD3 and GAD5 probably are probably not regulated by Ca^++^/CAM [14]. In Arabidopsis, GAD1 is expressed primarily in the root [15].

The regulation of GABA itself has also been investigated. It has been suggested that nitric oxide [16] might regulate GABA levels. It has also been suggested that GABA might be involved in mitigating ROS in plants and that the GABA shunt pathway plays could play a role either as metabolites or endogenous signaling molecules in multiple regulatory mechanisms under stress [17]. The functions and regulation of GABA has been reviewed in other recent publications [18–20].

Several approaches have been used to manipulate GABA levels and metabolism to study its function in plants. GAD1 has been decoupled from its normal regulation by Ca^++^ and CAM by the removal of its C-terminus and then subsequently over-expressed in plants [3, 20]. GABA pathway and transport genes have been both over-expressed and suppressed in plants [3, 20]. Inhibitors of GAD have been used to reduce GABA levels [21]. In addition, targeted mutagenesis has been used to increase GABA content in tomato fruits [22] and rice [23], albeit, in the case of tomato, for a nutritional purpose. For an example of the over-expression approach, transgenic tobacco plants mis-expressing a mutant petunia GAD lacking the C-terminal CAM binding domain have high GABA levels but exhibit severe abnormalities showing that Ca^++^ and calmodulin regulate GAD activity in plants [24].

The expression of other genes involved in the GABA shunt or GABA metabolism has also been manipulated. For example, although the over-expression of GABA-AT (GABA aminotransferase) in Arabidopsis had no effect on phenotype in normal growing conditions [25], it reduced the low-temperature induced increase in GABA concentrations; whereas GABA-AT mutants accumulate GABA under normal conditions and have fewer seeds [26].

In this study, we have taken two of these approaches to manipulate GABA metabolism in *Zea mays*. First, we have produced transgenic maize plants constitutively the wild-type and the deregulated forms of AtGAD1 to test the effect of increasing GABA levels. In addition, we have suppressed several GABA pathway or transport genes to decrease GABA, manipulate its accumulation or enhance flux through the GABA shunt.

## Materials and methods

### Transgenic studies on truncated GAD1

A deregulated *Arabidopsis* GAD1 sequence (AT5G17330 lacking amino acids 457 to 502) under rice RTBV promoter [27] was transformed into corn (Zea mays L) using *Agrobacterium tumafaciens* system [28]. Independent transformation events were selected and advanced to be marker free and single copy by the method described in Rice et al. 2014 [29]. In brief, immature embryos were excised from a post-pollinated maize ear. After co-culturing the excised immature embryos with Agrobacterium carrying the AtGAD1-ΔCaMD plasmid vector, the immature embryos were placed on selection medium containing glyphosate and carbenicillin disodium salt in order to inhibit the growth of untransformed plant cells and excess Agrobacterium. To confer tolerance to glyphosate the AtGAD1-ΔCaMD plasmid vector also contained the selectable marker CP4 EPSPS coding region regulated by rice actin promoter, leader, and intron, and a 3′ polyadenylation sequence. Events containing the AtGAD1-ΔCaMD cassette were characterized by detailed molecular analyses to select events that contain only one copy of the AtGAD1-ΔCaMD cassette. The early phenotypic observations were conducted on 30-day-old R0 transformants under greenhouse growth conditions. Lyophilized leaf samples from five individual R0 transformants with severe phenotypic defects were analyzed for changes in free amino acids.

### GABA pathway gene suppression in corn

Corn genes from the GABA biosynthesis, plasma membrane transport and GABA shunt pathway (Table 1) were suppressed using artificial miRNA technology [30]. Binary constructs with the synthetic miRNA sequence downstream of a constitutive DaMV promoter was transformed into corn (Zea mays L) using *A*. *tumafaciens* transformation system [28]. Lines were selected and maintained by the method described for the truncated AtGAD1. The R0 transformants were monitored for phenotypes. Transgenic lines that reached normal reproductive development were crossed with themselves and R2 homozygotes were selected for phenotype confirmation and further molecular and metabolic analysis. Multiple lines were tested for each GABA pathway gene.

**Table 1:** List of corn GABA metabolic pathway gene transformants and their targeted for suppression using synthetic miRNA.

Gene name	Gramene ID	GABA metabolism	Synthetic miRNA	Target Sequence (nt)	
ZmGAD5	GRMZM2G	GABA biosynthesis	>TTGATGTGGAAGA	900..920	
	355906		TGAGCTCA		
ZmGAD4	GRMZM2G	GABA biosynthesis	>TTGTTCTGCCAT	423..443	
	017110		TCCTCTTA		
GABAP2	GRMZM2G	GABA transport	>TTGAAGGCGAGGT	978..998	
	022642		AGAACACA		
GAT	GRMZM2G	GABA transport	>TCATCATGAAGAC	506..526	
	154958		GCCGAAGC		
GABAT2	GRMZM5G	GABA shunt	>TTCTTCTTGTCTG	549..569 891209	GCCTTCCA
SSADH1	GRMZM2G	GABA shunt	>TCTTGCACCAATA	1005..1025	
	128114		TCCTGTTA		
GHBDH1	GRMZM2G	GABA shunt	>TTAACTGCCTCGC	306..326	
	153984		TAATCTTA		
SCoA	GI:195620	GABA shunt	>TTCAGTTCCACCTT	223..243	
alpha	061		TCTTGGA		
subunit					

GAD4, glutamate decarboxylase 4; GAD5, glutamate decarboxylase 5; GAT1, Cell membrane GABA transporter; GABAP, mitochondrial membrane GABA permease; SSADH, succinate-semialdehyde dehydrogenase; GHBDH1, γ-hydroxybutyrate (GHB) dehydrogenase; SCoA, succinyl CoA ligase subunit alpha; GABAT, GABA aminotransferase.

### RNA extraction and quantitative RT-PCR

RNA was extracted using the EZNA RNA Purification Kit (Omega BioTek, #r1027-02). RNA samples were then treated with Turbo DNA-Free DNase (Ambion) and normalized to 5ng/μL. Primers and probes for qRT-PCR were selected using Applied Biosystems Primer Express version 2.0 software (S1 Fig). qRT-PCR was carried out using the TaqMan® One-Step RT-PCR Master Mix Reagents Kit (Applied Biosystems Cat# 4309169) in an ABI7900HT following Applied Biosystems manufacture recommendations with a 60°C annealing temperature and 40 cycles. A final primer concentration of 300 nM and 200 nM probe for was used for each reaction. For 18S, a final primer concentration of 100 nM and 100 nM probe was used. Comparative gene expression (2^-ddCt) was used for data analysis using 18S for normalization. (see ABI Prism 7700 Sequencing System User Bulletin #2).

### GAD extraction and assay

The extraction of GABA was similar to that described for Asn [31]. Briefly, each sample of fresh-frozen powdered plant tissue was dispensed into a single well of a deep-well 96-well plate along with two small ball bearings to facilitate extraction. A 4:1 (v/w) mixture of GAD extraction buffer (20% glycerol, 1 mM DTT, 1 mM EDTA, and 50 mM HEPES-KOH, pH 7.5) was used for extraction. The plate was then shaken at top speed in a MEGA grinder for 3 min. The resulting slurry was clarified by centrifugation at 4000 RCF and the supernatant was then desalted into the extraction buffer using a 96 well G-25 spin plate (SNS 025L; The Nest Group). The protein extract was then analyzed for activity or frozen at -80°C for later assay. Plant GAD is relatively stable through at least one quick freeze/thaw cycle.

GAD activity was assayed in a 96-well plate. For routine measurements, a small amount of crude extract (1–5 μL) of desalted extract was added to 100 μL of 1 mM CaCl2, 1 mM PLP, 1 mM DTT, 20 mM Glutamate, 31.25 μg Calmodulin from Bovine Testes (Sigma, P1431) for 30–60 min and then the reaction was stopped by the addition of 100 μL 10% TCA and the samples clarified by centrifugation. The amount of GABA was determined on an HPLC as described for asparagine [31] and was linear with respect to incubation time and protein concentration. For the plant measurements, a construct level analysis of multiple events was performed with n greater than or equal to 3. ANOVA analysis was performed in GraphPad 7.0.

### Extraction and analysis of free amino acids

GABA and primary free amino acids were analyzed using HPLC after derivatization with o-phthalaldehyde / 9-fluorenyl-methyl chloroformate (OPA/FMOC). Briefly, 30 mg of lyophilized leaf tissue was extracted with 1mL of 5% (v/v) trichloroacetic acid. The supernatant was injected onto an Agilent 1100 HPLC and the column eluent was monitored using fluorescence at an excitation/emission wavelength of 340/450 nm, respectively. Online pre-column derivatization with OPA was performed using the autosampler. Separation was performed on a ZORBAX Eclipse-AAA column (4.6 x 150 mm) using the solvent systems A: 40 mM NaH2PO4 pH 7.8 and solvent B: Acetonitrile: Methanol: water (45:45:10, v/v/v) and a gradient program of 5% B to 100% B in 12 min. GABA standard was purchased from Sigma Aldrich (part number A2129), and primary free amino acids were quantified using standard calibration curves. All amino acid standards (Agilent part number 5061–3330) and derivatization reagents were obtained from Agilent, (part numbers borate buffer 5061–3339; OPA 5061–3335, and FMOC 5061–3337)

## Results and discussion

### High levels of GABA retards vegetative growth causing tassel skeletonization and sterility

**Fig 2:**
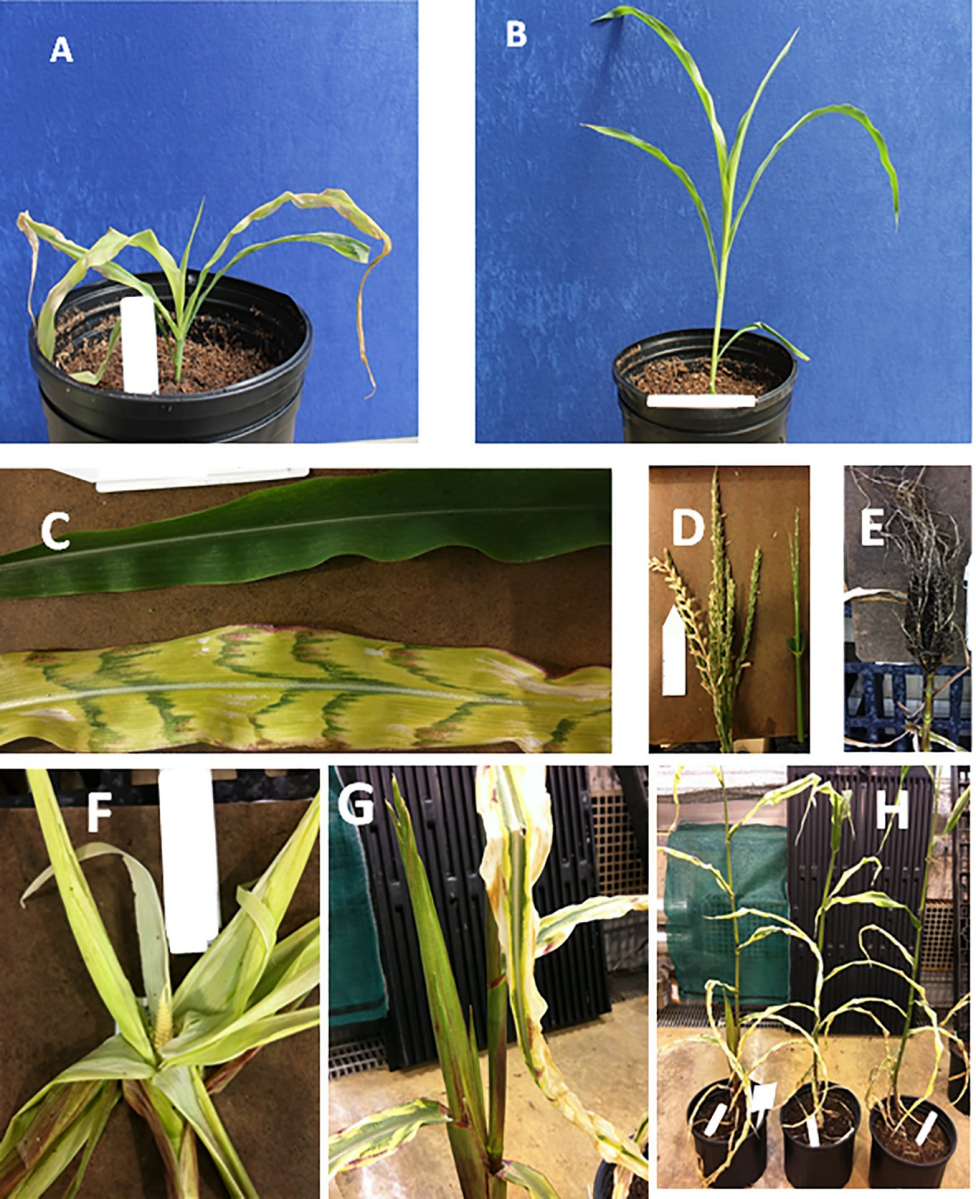
Growth deformities in AtGAD1-ΔCaMD plant expressed under RTBV promoter A. **A:** 30-day-old AtGAD1-ΔCaMD; **B:** 30-day-old wild-type, **C**: 150-day-old leaf of wild-type; 150-day-old leaf of AtGAD1-ΔCaMDt; **D:** 150-day-old tassel of AtGAD1-ΔCaMD; **E:** 150-day-old root of AtGAD1-ΔCaMD; **F:** 150-day-old ear of AtGAD1-ΔCaMD plant; **G:** 150-day-old ear of AtGAD1-ΔCaMD; H: 150-day AtGAD1-ΔCaMD entire plant.

R0 plants, thirty days after sowing, expressing deregulated AtGAD1-ΔCaMD gene under RTBV vascular-specific promoter showed severe leaf chlorosis, necrotic lesions, and stunting. The plants appeared pale green (Fig 2A, 2B). The adult transgenic plants (150-days-old) retained all the deformities observed during early stage of plant growth and showed other phenotypic defects such as reduced plant height (Fig 2A), Band leaf size, variegations on the leaf lamina and a complete lack of reproductive development (Fig 2C–2H). A few R0 plants that transitioned to reproductive stage manifested severe tassel, ear deformities and sterility (Fig 2).

**Fig 3:**
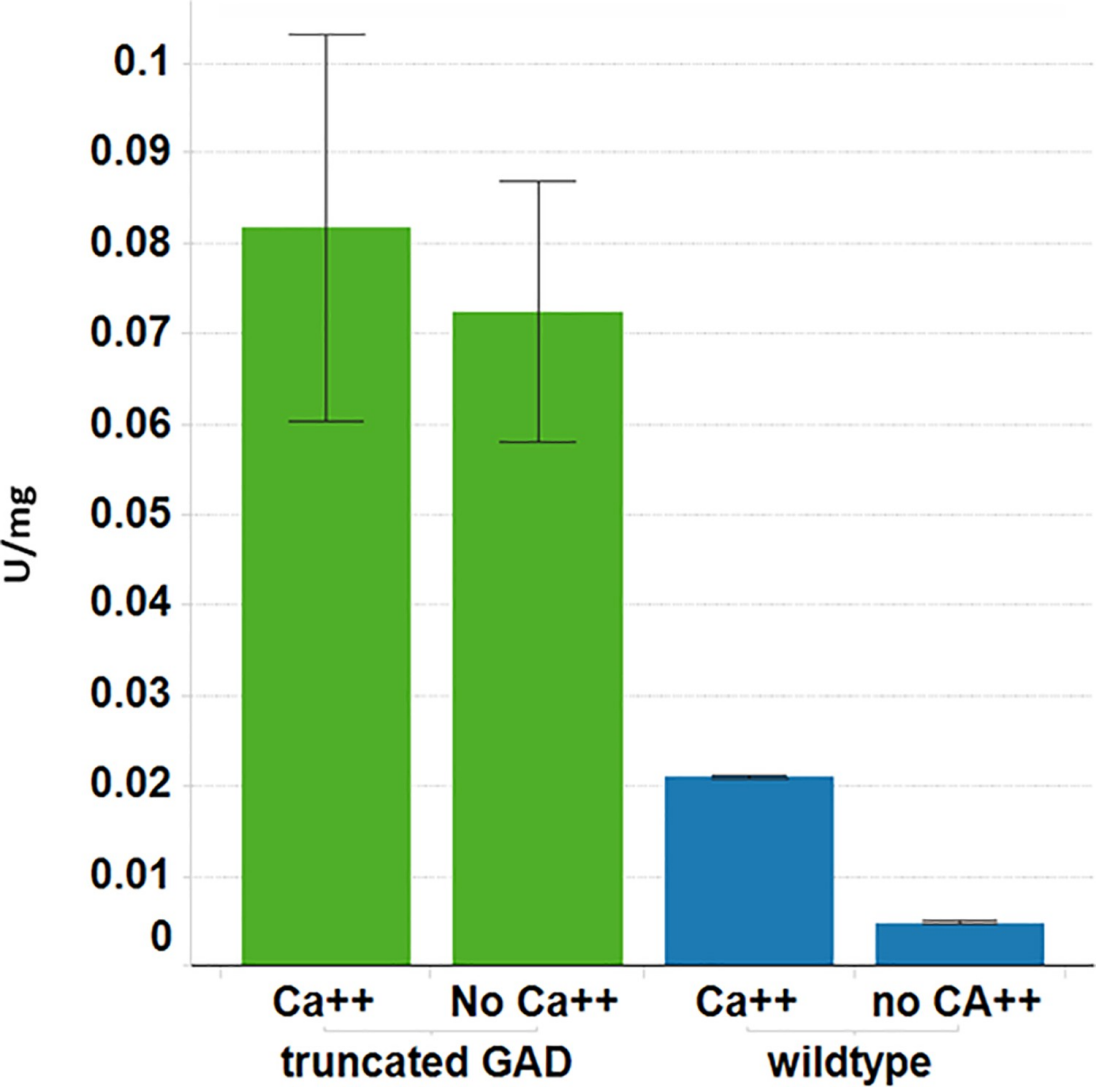
GAD enzyme activity in the leaves of 150-day-old plants over-expressing a truncated GAD with or without 10 μM Ca^++^/10 ng/μL Calmodulin. Values are the average of 5 events and the error bars represent standard error from the mean. Green bars represent transgenic plants, blue bars represent wild-type plants.

GAD activity in leaf tissue harvested from one-month old R0 plants expressing a truncated AtGAD1 lacking the C-terminal CAM binding domain was at least 4-fold higher than in wild-type plants and as expected was insensitive to Ca^++^/CAM (Fig 3).

**Fig 4:**
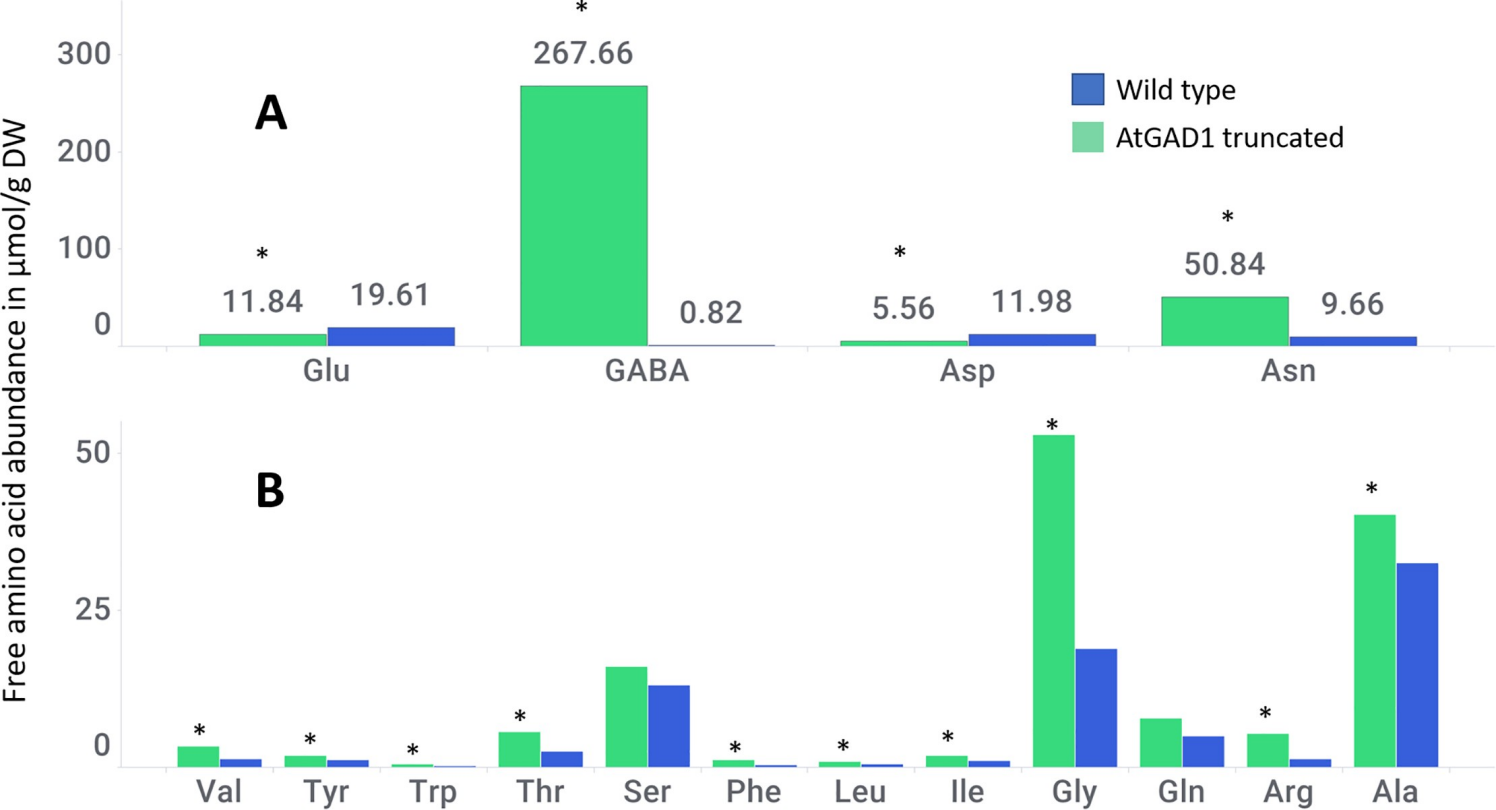
Truncated AtGAD1-ΔCaMD expressing transgenic 150-day-old corn plants show increased accumulation of GABA and other amino acids. Values are the average of five events. Green bars represent transgenic plants, blue bars represent wild-type plants. An asterisk marks those lines significantly different than wild type (p < 0.05).

Free amino acid (FAA) analysis of leaf tissues from the 30-day-old R0 plants revealed that γ-aminobutyrate (GABA) levels were ~320-fold higher and asparagine (Asn) was ~5 fold higher than wild-type control plants (Fig 4) which is consistent with other reports which show that GABA levels can be increased by manipulating GAD levels which also leads to changes in the other amino acids [23, 32, 33], and that GABA levels are primarily controlled through manipulating GAD levels. Concomitantly, the abundance of glutamate and aspartate, the precursor amino acids for GABA and Asn were reduced by 1.6- and 2-fold, respectively (Fig 4) consisted with observations of Takayama et al. 2017 in tomato fruit who removed the autoinhibitory domain of tomato GAD3 [32]. Most other amino acids except for glutamine and serine were significantly higher in the truncated plants than the wild-type control plants (p<0.05). The FAA data indicates that the observed phenotype may not be a consequence of general amino acid starvation. Future metabolite profiling studies could help determine the basis of plant fitness cost associated with GABA overproduction.

### Suppression of GABA biosynthesis does not affect growth under standard growing conditions

**Fig 5:**
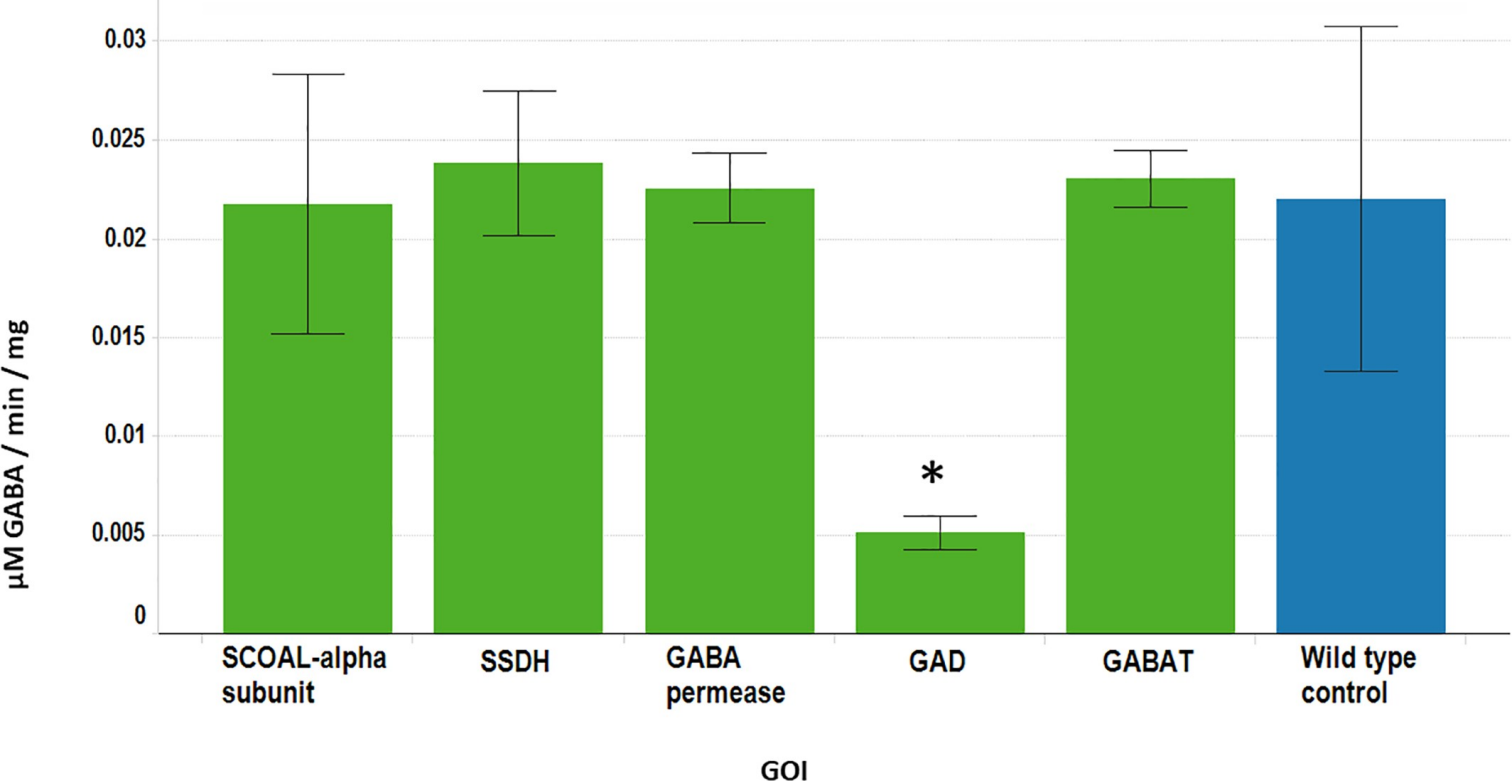
GAD activity in the leaves of GABA pathway suppression mutants. The values represent means of duplicate measurements of 5 separate plants. Green bars represent transgenic plants, blue bars represent wild-type plants. An asterisk marks those lines significantly different than wild type (p < 0.05).

**Fig 6:**
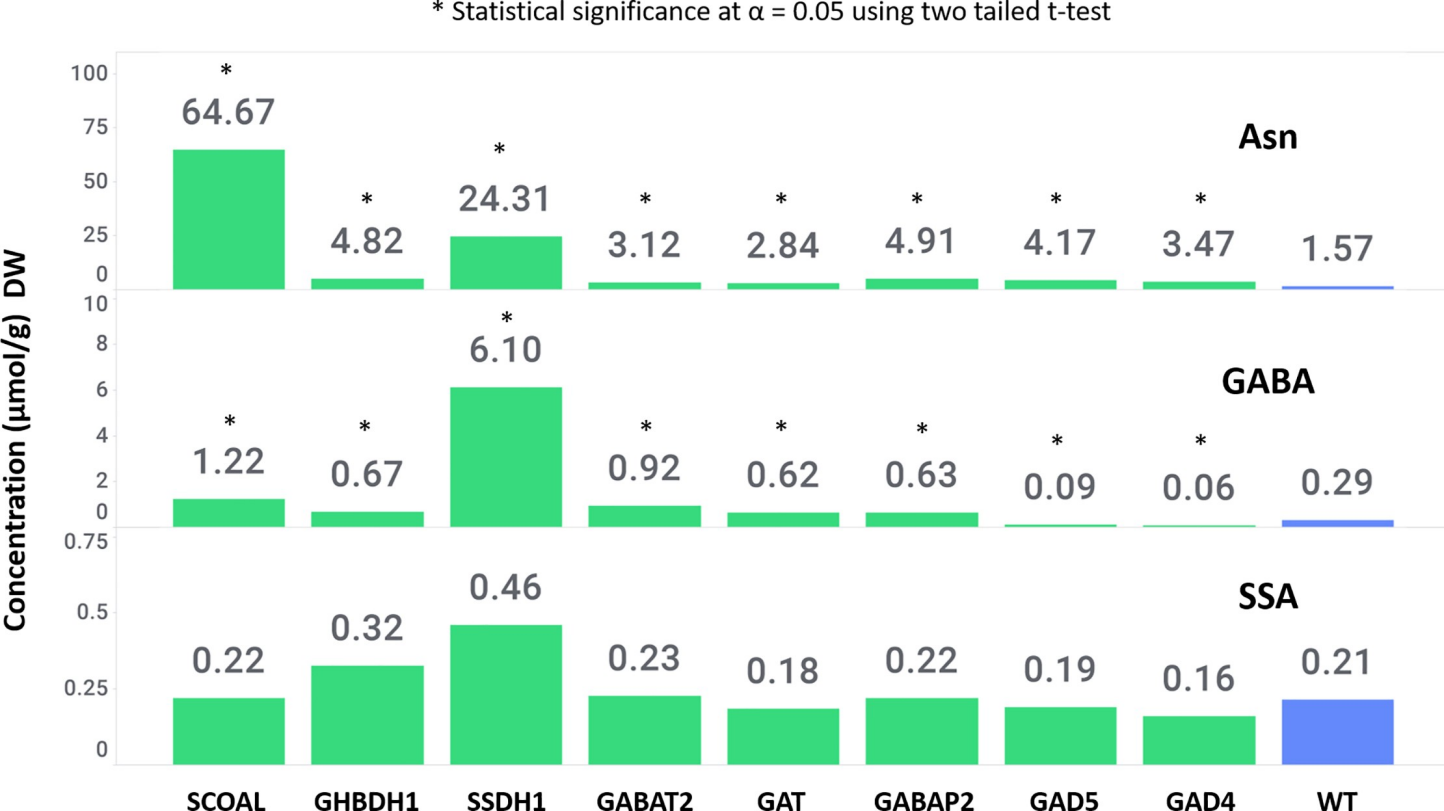
GABA and SSA levels in the leaves of GABA pathway suppression mutants. The values represent means of duplicate measurements of 5 separate plants. Green bars represent transgenic plants, blue bars represent wild-type plants. An asterisk marks those lines significantly different than wild type (p < 0.05).

Independent corn transgenic suppression lines with constitutively lower level of GAD4, GAD5 GABA biosynthetic gene expression or GABAP and GAT genes involved in GABA transport expression showed normal growth and development under standard growth conditions. A 3.3-fold reduction in GABA levels and a 4-fold lower GAD enzyme activity was observed in the GAD5 suppression line (Figs 5 and 6). Suppression of GABA transporter gene expression was not found to decrease GABA levels or GAD activity, under the conditions tested in this study (Figs 5 and 6), and had no perturbation of the amino acid levels except for 2-3-fold higher levels of Asn in biosynthetic suppression lines compared to the WT.

### Suppression of GABA shunt pathway compromises plant growth and reproductive development

Independent corn transgenic suppression lines with constitutively lower level of the GABA catabolic pathway gene GABAT did not cause growth and developmental defects. Suppression of GABAT had no effect on GAD activity and these plants had higher levels of GABA and Asn by 3 and 2-fold respectively compared to WT. No change in the SSA level was observed as no perturbations higher than 2-fold were observed in the abundance of other amino acids in the GABAT suppression line. Similarly, suppression of GHBDH resulted in slight 2-fold increase in only GABA and Asn levels compared to the WT.

**Fig 7:**
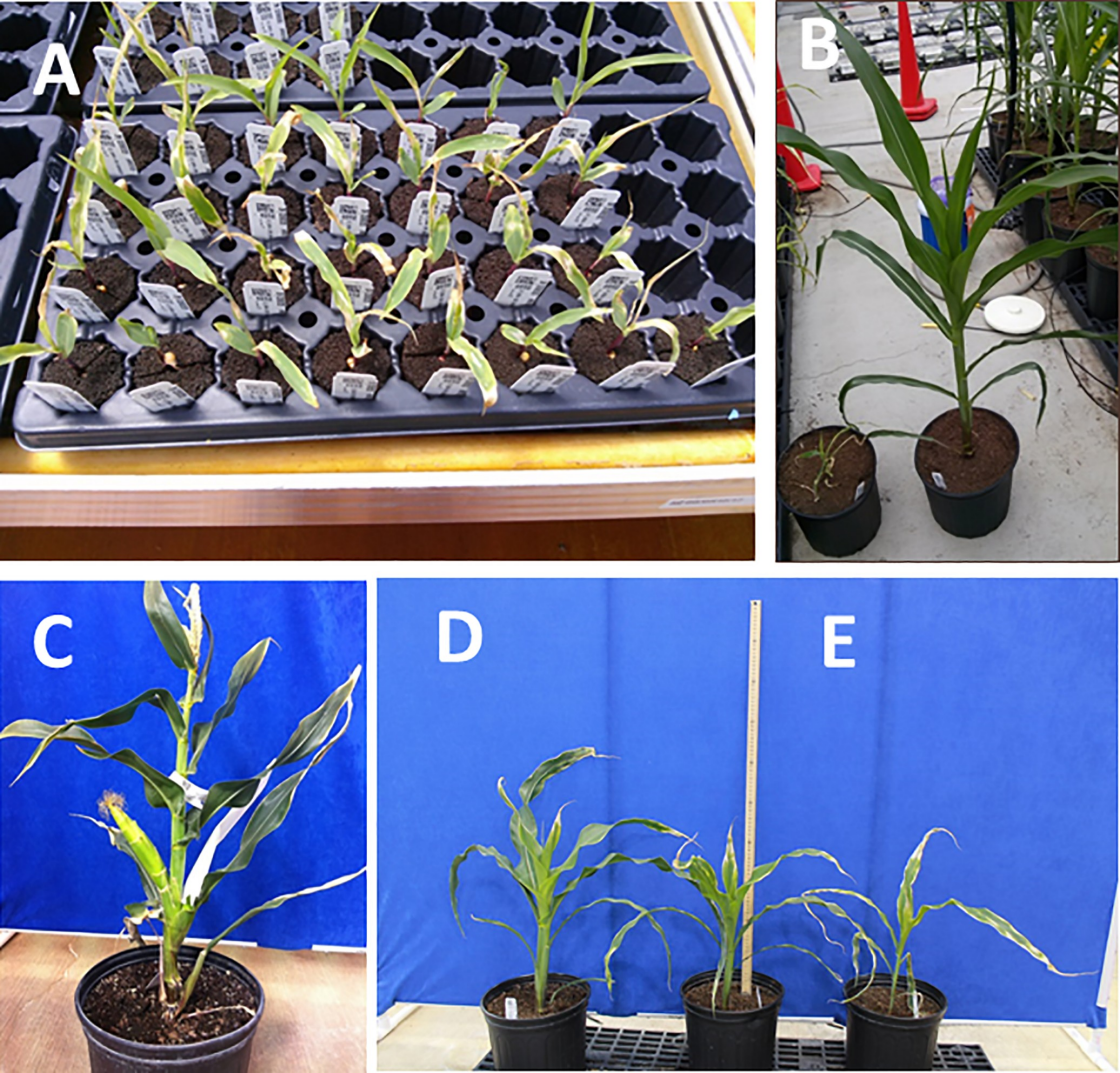
Growth deformities upon perturbation of GABA shunt pathway. **A**: SSADH 10-day-old seedlings; **B**: 30-day-old, SSADH on the left, wild-type on the right; **C**: 60-day-old SSADH plant; **D**: Wild-type plant on the left, two SCoAL plants on the right.

The suppression of the SSADH gene caused lethality in homozygous R1 lines, whereas in hemizygous lines (R0 and R1) severe growth defects and leaf variegations without affecting reproductive growth and development were observed (Fig 7). Similarly, suppression of the SCoAL alpha subunit gene, expected to function in the Krebs cycle and GABA shunt pathway caused plant growth retardation accompanied by failure to transition to the reproductive phase of development (Fig 7). GAD enzyme activity was not perturbed in these lines (Fig 5), while GABA accumulated to levels that is 20- and 4-fold higher than the WT in SSADH and SCoAL respectively (Fig 6). SSA, the product of GABA shunt was only higher in SSADH suppression line as expected with 2.0-fold increased than WT (Fig 6). Several amino acids levels were perturbed in both lines showing accumulation of Gly, Phe and specially Ser and Glu in SCoAL suppression line (S1 Table). However, Asn levels was the highest accumulated amino acids in both lines with levels at 15- and 40-fold higher than the WT for SSADH and SCoAL respectively (Fig 6).

### GABA has a role in controlling plant growth and development which is conserved across monocot and dicots

Based on the results, we conclude that overexpression of deregulated GAD1 leads to the off-type phenotype and a buildup of GABA in maize. There is no observable reduction in total free amino acids leading to amino acid starvation in these transgenic plants. It needs to be determined if the phenotypic abnormalities are a consequence of build-up of aldehyde intermediates such as SSA compromising plant growth and development. Akama and Takaiwa, [33], reported that rice calli derived from embryos expressing OsGAD2 (OsGAD2ΔC) under the control of the CaMV promoter showed a 100-fold increase in GABA relative to wild type control. All the other amino acids tested were lower than the wild-type control. Taken together the data indicates that the amino responses of maize to GAD over-expression differed from those previously observed in rice. However, the overexpression of deregulated GAD from rice and other dicot plants had aberrant phenotypes such as stunting chlorotic leaves and sterility [23, 33] similar to that observed in maize in this study and that changes in aldehyde intermediates as well as important amino acids such as glutamate and asparagine are indicative of a carbon/nitrogen imbalance in maize [34, 35]. A GABA shunt enzyme succinic semialdehyde dehydrogenase is involved in patterning of Arabidopsis leaves [36]. In addition, the observed growth deformities is in general accordance with the role of GABA as a signal molecule controlling plant growth.

Apart from SSADH, other GABA metabolic pathway suppression lines did not show abnormalities under standard growth conditions. Mutation in enf1 (SSDH) leads to abaxialized and adaxialized leaves and this phenotype is suppressed by an additional mutation in GABA-AT [37]. Bouche et al. [37] reported that the loss of function Arabidopsis SSADH knock-out plants (At1g79440) had greater sensitivity to oxidative damage due to hyper-accumulation of ROIs (Reactive Oxygen Intermediates) under light and heat stress. Bouche et al. 2003 [38] demonstrated that the GABA shunt had a role in restricting levels of restricting ROS levels in plants and Ansari et al. 2021 [17] reviewed the role of GABA in mitigating ROS stress in plants. We did not test stress conditions, but our results indicate that SSADH suppression lines show abnormal growth phenotype under standard growth conditions and as expected they accumulate SSA. It is intriguing that these plants also accumulate higher levels of GABA which could be a metabolic adjustment to regulate GABA transport into mitochondria to overcome the dysfunction in GABA catabolism, in this case the plants may respond to reduced shunt flux by increasing GABA levels in an attempt to reverse that process. Vegetative anomalies have been shown to accompany increases in GABA levels in some cases [5]. However, given the abnormalities we observed in the plant which expressed truncated GAD it is possible that the developmental and growth abnormalities of SSDH suppression lines could also be due to over production of GABA and the accumulation of SSA with the likelihood of reduced succinate supply to the TCA cycle through anapleurotic GABA shunt channel. In any event, A host of abnormal metabolite profiles have been linked to disruption of TCA cycle [3, 5] The growth defects of SCoAL suppression lines in our study lend credence to the primary role of TCA cycle in supporting plant growth and development and that, secondary routes of succinate replenishment through GABA shunt is incapable of supporting normal plant growth even under standard growth conditions, in corn. By contrast, tomato suppression lines with drastic reduction in SCoAL activity showed mild vegetative growth effects with significant reduction in leaf biomass, fruit yields and a compensatory increase in GABA shunt mediated succinate production [39].

In conclusion, our results indicate that the function of GABA and GABA shunt pathway in regulating plant growth and reproductive development under standard growth conditions is conserved across monocots and dicots, although species specific differences exist at the metabolic level. Enhancing GABA levels in a non-specific manner has a profound effect on plant growth and development. However, effects of suppressing other GABA-related genes involved in the GABA shunt and GABA transport may be more subtle, primarily effecting the non-housekeeping (stress-related) functions. Additional profiling studies are required to help expand our understanding of molecular and metabolic crosstalk that links GABA with plant growth, development, carbon partitioning and stress adaptation.

## Supporting information

S1 Fig Suppression construct mRNA expression data. Suppression of each construct was measured as described in the Materials and Methods: A, ZmGAD4 family; B, SSDH; C, GAT; D, GHBDH; E, SCOAL; F, ZmGAD3; G, GABAT; H, GAD5. Values are the average of 5 events and bars represent standard error from the mean.
(TIF)

S1 Table Amino acid levels in GABA pathway suppression lines (ppm). Abbreviations used in this table are defined in the legend of Table 1.
(XLSX)
