# Supplementary figures and images for: Phenotypic effects from the expression of a deregulated AtGAD1 transgene and GABA pathway suppression mutants in maize
Source: PLoS One. 2021 Dec 6;16(12):e0259365. doi: 10.1371/journal.pone.0259365 (PMC8648116; doi:10.1371/journal.pone.0259365)

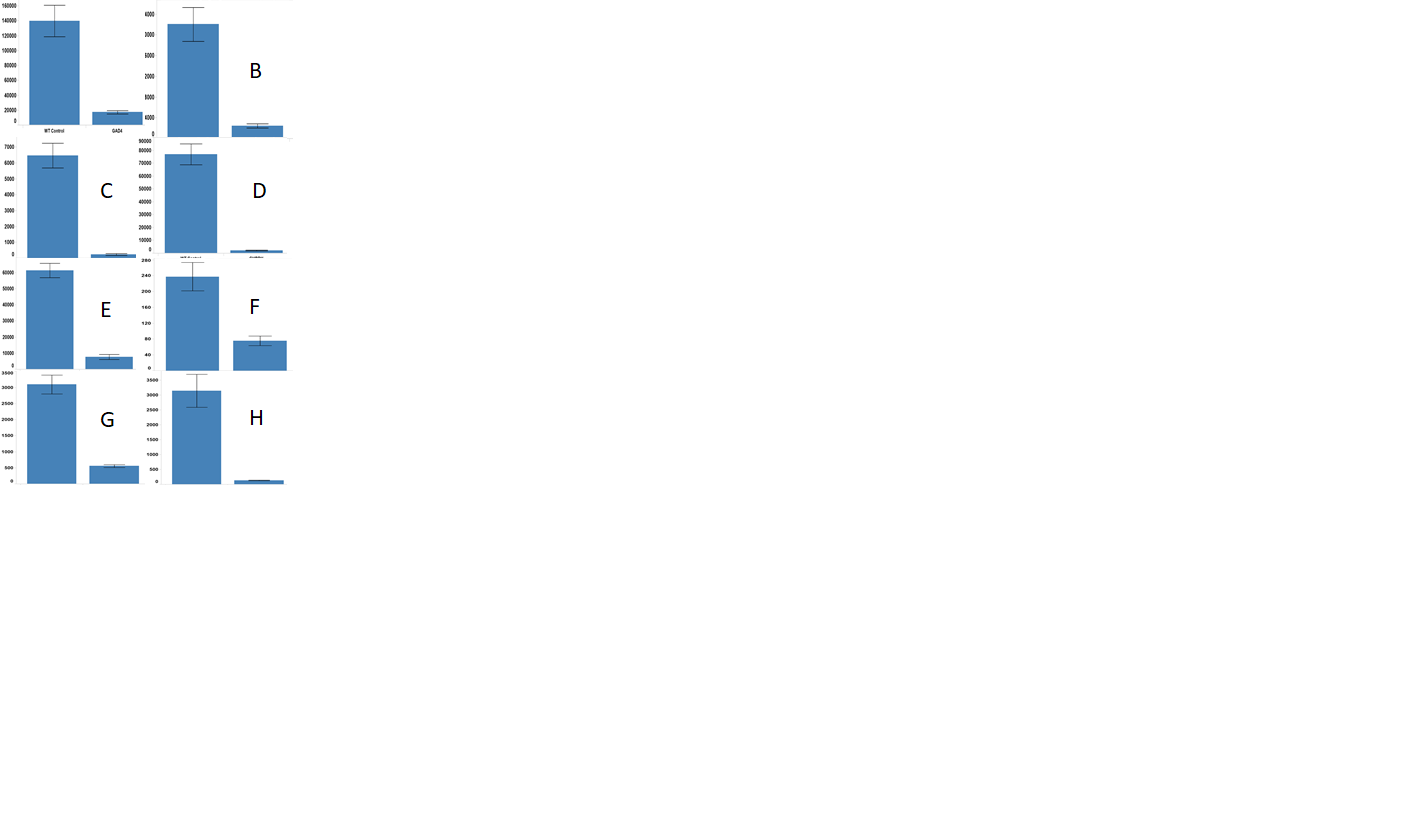

Supplement: S1 Fig — Suppression of each construct was measured as described in the Materials and Methods: A, ZmGAD4 family; B, SSDH; C, GAT; D, GHBDH; E, SCOAL; F, ZmGAD3; G, GABAT; H, GAD5. Values are the average of 5 events and bars represent standard error from the mean. (TIF) [file pone.0259365.s001.tif]
